# Supplementary material for: Dynamic layer-specific processing in the prefrontal cortex during working memory
Source: Commun Biol. 2024 Sep 14;7:1140. doi: 10.1038/s42003-024-06780-8 (PMC11401931; doi:10.1038/s42003-024-06780-8)
Supplement: Supplementary file 2 — Supplementary material [file 42003_2024_6780_MOESM2_ESM.pdf]

Supplementary Figures

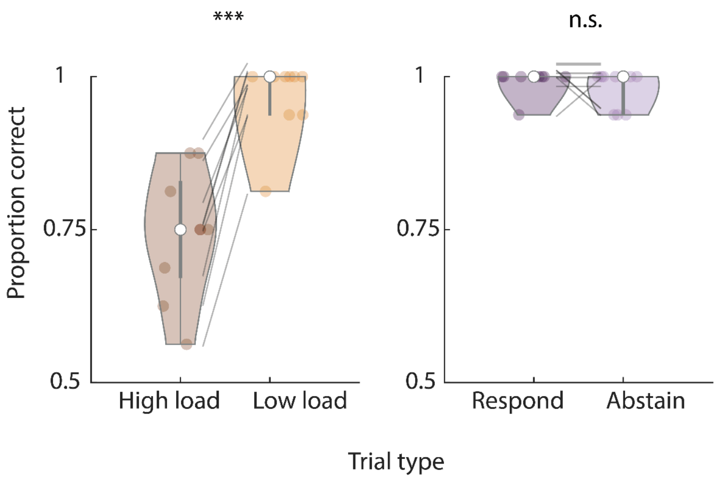

Supplementary figure 1: Behavioral responses. The left panel shows the accuracy of subjects in the high and low load trials. The right panel shows the accuracy of responding or abstaining to respond in motor trials. Colored points depict individual participants; white points depict the mean. Error bars indicate ±SD.

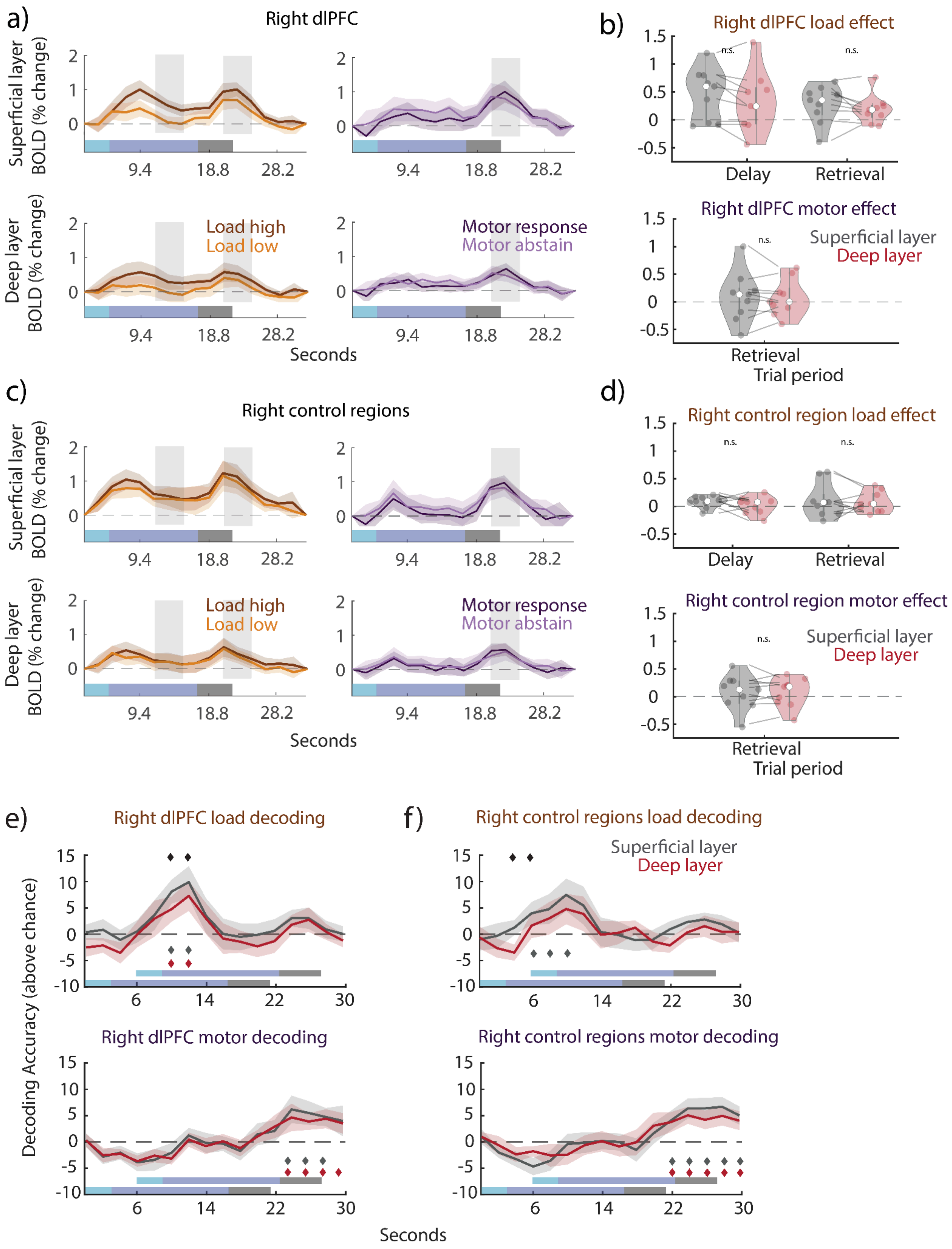
a)
Right dlPFC
Superficial layer BOLD (% change)
Deep layer BOLD (% change)
Load high
Load low
Motor response
Motor abstain
9.4
18.8
28.2
Seconds
b)
Right dlPFC load effect
n.s.
Delay
Retrieval
Right dlPFC motor effect
Superficial layer
Deep layer
Retrieval
Trial period
c)
Right control regions
d)
Right control region load effect
Right control region motor effect
e)
Right dlPFC load decoding
Right dlPFC motor decoding
Decoding Accuracy (above chance)
f)
Right control regions load decoding
Right control regions motor decoding
Seconds

Supplementary figure 2: Layer-specific univariate BOLD time courses and multivariate decoding in the right hemisphere. a-d) Same as **Fig. 2**, but for right dlPFC and right frontal control regions. Load effect between layers during delay in the right dlPFC: $p = 0.071$, $t(8) = 2.08$, $CI^{95} = [-0.014, 0.27]$, $d = 0.69$, two-tailed t-test. Load effect between layers during the retrieval period in the right dlPFC: $p = 0.24$, $t(8) = 1.27$, $CI^{95} = [-0.073, 0.25]$, $d = 0.43$. Motor effect between layers during retrieval in the right dlPFC: $p = 0.75$, $t(8) = 0.332$, $CI^{95} = [-0.12, 0.16]$, $d = 0.11$. Load effect between layers during the delay period in the right control region: $p = 0.22$, $t(8) = 1.339$, $CI^{95} = [-0.046, 0.17]$, $d = 0.45$. Load effect between layers during the retrieval period in the right control region: $p = 0.66$, $t(8)= 0.45$, $CI^{95} = [-0.12, 0.18]$, $d = 0.15$. Motor effect between layers during retrieval in the right control region: $p = 0.99$, $t(8) = 0.01$, $CI^{95} = [-0.09, 0.09]$, $d = -0.004$. **e-f)** Same as **Fig 3 a-b)**, but for right dlPFC and right frontal control regions. Right dlPFC superficial layer load decoding cluster at 10-12 s ($p = 0.0059$, $d_{cluster\ \bar{X}} = 1.17$, one-tailed permutation test); deep layer cluster at 10-12 s ($p = 0.045$, $d_{cluster\ \bar{X}} = 0.75$, one-tailed permutation test). Between layer load decoding cluster at 10-12 s ($p = 0.039$, $d_{cluster\ \bar{X}} = 1.35$, two-tailed permutation test). Right control region superficial layer load decoding cluster at 6-10 s ($p = 0.013$, $d_{cluster\ \bar{X}} = 0.71$, one-tailed permutation test). Between layer load decoding cluster at 4-6 s ($p = 0.030$, $d_{cluster\ \bar{X}} = 1.11$, two-tailed permutation test). Right dlPFC superficial layer motor decoding cluster at 24-28 s (p = 0.0013, $d_{cluster\ \bar{X}} = 0.95$, one-tailed permutation test); deep layer cluster at 24-30 s ($p < 0.001$, $d_{cluster\ \bar{X}} = 0.99$, one-tailed permutation test). Right control region superficial layer motor decoding cluster at 22-30 s ($p < 0.001$, $d_{cluster\ \bar{X}} = 1.24$, one-tailed permutation test); deep layer cluster at 22-30 s ($p < 0.001$, $d_{cluster\ \bar{X}} = 1.18$, one-tailed permutation test).

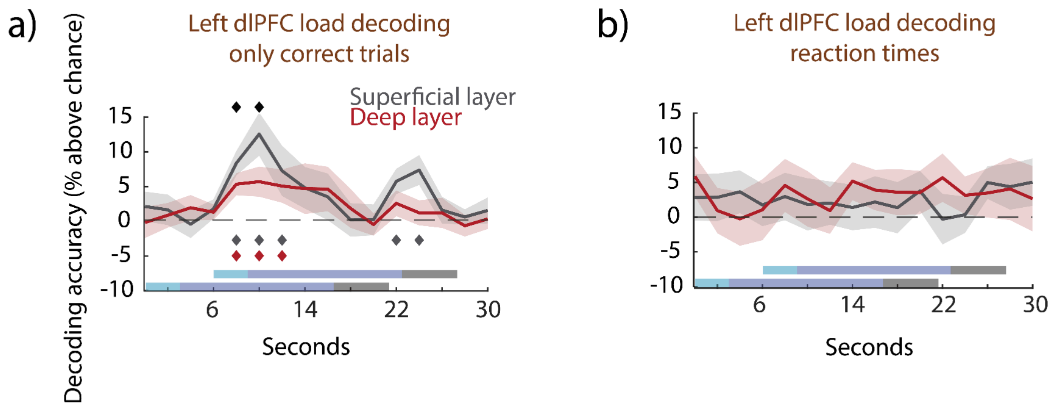

Supplementary figure 3: Layer-specific multivariate decoding in the left dlPFC accounting for accuracy and reaction time . a) Multivariate decoding analysis of load when accounted for accuracy. We removed all trials where the response was incorrect and ran the multivariate decoding on the remaining correct trials. We find two above-chance clusters in the superficial layers during the encoding (p = 0.0003, $d_{cluster\,\bar{X}}$ = 1.18, one tailed permutation test) and retrieval periods (p = 0.0051, $d_{cluster\,\bar{X}}$ = 1.15, one tailed permutation test); one cluster in the deep layers (p = 0.002, $d_{cluster\,\bar{X}}$ = 0.92, one tailed permutation test); and one cluster between superficial and deep (p = 0.014, $d_{cluster\,\bar{X}}$ = 1.50, two-tailed permutation test). **b)** Multivariate decoding of high and low reaction times. We calculated the median reaction time of high and low load trials separately, and then labeled the above-median high and low load trials as one class and the below-median high and low load trials as the other class. Trials where no response was made were excluded. We find no significant clusters.

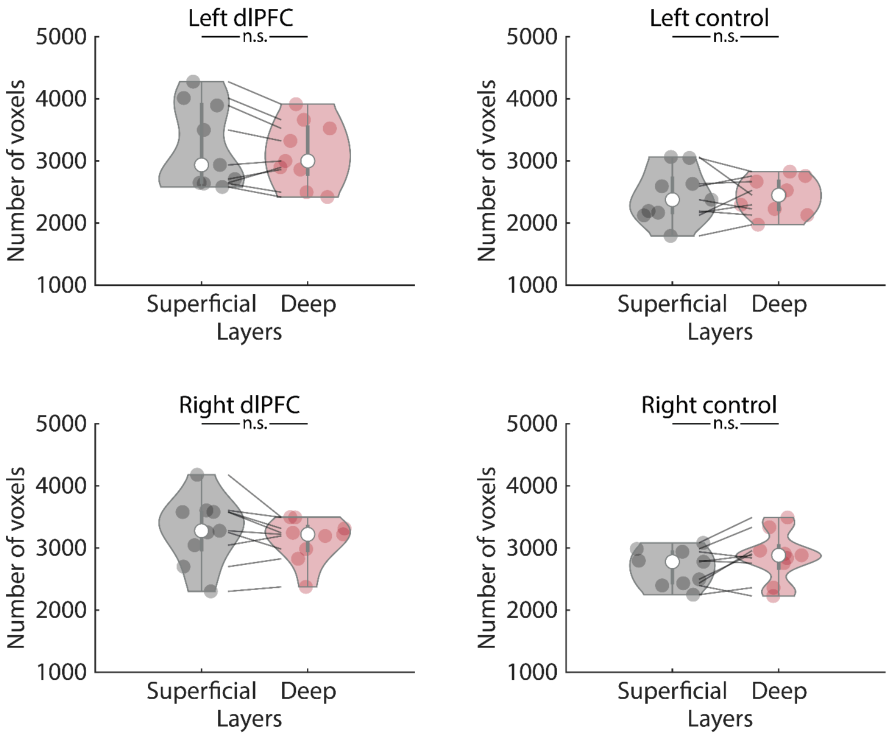

Supplementary figure 4: Number of voxels per layer per ROI. We find no significant difference in the number of voxels between layers in the left dlPFC (p = 0.15, $t(8)$ = 1.582, CI^{95} = [-55.7, 299], d = 0.53), left control region (p = 0.88, $t(8)$ = 0.157, CI^{95} = [-211, 241], d = 0.05), right dlPFC (p = 0.13, $t(8)$ = 1.72, CI^{95} = [-52.5, 358], d = 0.57), right control region (p = 0.068, $t(8)$ = 2.109, CI^{95} = [-373, 16.6], d = -0.70).

Supplementary Discussion

Our fMRI effect size in terms of percent signal change appears to be considerably lower when compared to Finn et al. [1] Whilst they were able to show robust motor effects in a smaller sample size in their original study (N=6) [2], we acknowledge that our sample is smaller than their final study (N=15). Consequently, we acknowledge that increasing the number of subjects in our study could theoretically reveal a significant motor effect in the deeper layers. However, a recent pre-registered direct replication attempt of Finn et al. [1] with 21 subjects found no higher activation of deep compared to superficial layers during the motor manipulation; neither in VASO nor GE-BOLD contrasts (Chaimow et al. *in prep*). Given these mixed results, further data should be acquired to resolve what cognitive operation might drive the deep layer responses in the dlPFC.

With respect to other potential confounding factors, we showed that layer-specific load decoding was not affected by accuracy or reaction time. Equally, we can reasonably exclude the possibility that uncertainty varied significantly between the two load conditions and could have contributed to a differential decoding of load in the two layers, as participants knew how many items they needed to remember at the beginning of the trial. Response conflict also did not drive decoding as in all load trials participants had the options of pressing one of two buttons. Whilst we did ask participants to fixate their gaze on the fixation cross, we did not record eye-tracking and, therefore, cannot rule out the effect of eye movements on load decoding. However, Bastos et al. [3] examined WM-related activity in the prefrontal cortex with laminar electrodes in monkeys and showed a robust deep layer activation processing saccades in the dorsolateral prefrontal cortex. We cannot rule out the possibility for decoding signals related to attentional load and the difference in processing four vs. one item is probably driving the decoding difference during the encoding period. However, this allowed us to investigate the laminar nature of where this type of load is decoded. Additionally, there may be potential confounds (e.g., increased tiredness, decreased attention) due to our participants performing the motor runs at the end of the scanning session, unlike the alternating run design used in Finn et al. [1] However, we believe the effects of differences in run order are negligible to our results, as subjects were performing at ceiling level in the motor runs, and the dlPFC's engagement appears to be robust at that trial stage, as evidenced by both univariate and multivariate analyses.

Supplementary References

1. Finn, E.S., Huber, L., Jangraw, D.C., Molfese, P.J., and Bandettini, P.A. (2019). Layer-dependent activity in human prefrontal cortex during working memory. Nat. Neurosci. *22*, 1687–1695. https://doi.org/10.1038/s41593-019-0487-z.
2. Finn, E. S., Huber, L., Jangraw, D. C. & Bandettini, P. A. Layer-Dependent Activity in Human Prefrontal Cortex during Working Memory. https://www.biorxiv.org/content/10.1101/425249v1.full (2018).
3. Bastos, A.M., Loonis, R., Kornblith, S., Lundqvist, M., and Miller, E.K. (2018). Laminar recordings in frontal cortex suggest distinct layers for maintenance and control of working memory. Proc. Natl. Acad. Sci. *115*, 1117–1122. https://doi.org/10.1073/pnas.1710323115.
